# Optical fibre Fabry-Pérot interferometer based on inline microcavities for salinity and temperature sensing

**DOI:** 10.1038/s41598-019-45909-2

**Published:** 2019-07-02

**Authors:** Raquel Flores, Ricardo Janeiro, Jaime Viegas

**Affiliations:** Department of Electrical and Computer Engineering, Khalifa University of Science and Technology, Masdar City Campus, Abu Dhabi, United Arab Emirates

**Keywords:** Fibre optics and optical communications, Optical sensors, Micro-optics

## Abstract

This work explores the development of highly sensitive salinity sensors. The demonstrated sensors are based on optical fibres and consist on Fabry-Pérot optical cavities formed by optimized processes that include chemical etching and fusion splicing, on which microfluidic channels are milled by focused ion beam. Two configurations are presented and their performance compared, including a design that makes use of Vernier-effect for the simultaneous measurement of salinity and temperature with high sensitivity. The interrogation of the devices is carried out by spectral measurements using a broadband light source yielding sensitivities to salinity up to 82.61 nm/M, or 6830.0 nm/RIU.

## Introduction

Salinity is a fundamental parameter and indicator of water quality, due to the effects it has on human health and other living organisms, with a large impact on the equilibrium of ecosystems. Commercially available solutions for measuring salinity are typically based on conductivity measurements. However, these systems are not immune to electromagnetic interference. In addition, the refractive index correlates to salinity better than conductivity^1,2^. Thus, optical fibre-based detection of salinity is a promising alternative offering many advantages such as immunity to electromagnetic interference, deployability, distributed and remote sensing, environmental friendliness, and cost-effectiveness^3^. Optical fibre-sensors for salinity find applications in varied environments, from industrial processes such as desalination plants, to marine environment. The *in situ* detection of salinity in marine environments through optical fibre probes has been demonstrated by several authors^2,4,5^.

Optical fibre-based sensors for salinity detection based on intrinsic refractive index (RI) changes include etched fibre-Bragg gratings^4^, surface plasmon resonance^6^, tapered optical fibres^7^, two-core fibres^8^, panda fibres^9^, and Fabry-Pérot (FP) interferometers^10^. For these sensing configurations, there is a trade-off between sensitivity and mechanical strength. Slight increases of the sensitivity have been demonstrated but at the cost of the sensor’s response time or mechanical strength. A good candidate to overcome such issues are interferometric sensors based on hollow cavities, in which the optical cavity is fabricated within the optical fibre. Hollow cavities within the optical fibre are typically fabricated by focused ion beam^10^, femtosecond laser ablation^11^, and chemical etching^12^. However, these techniques required either a long fabrication time, or complex fabrication processes. One other technique for the fabrication of hollow cavities, which has been growing in popularity, is through fusion splicing^13^, a low-cost and simple fabrication process, where the length of the cavity can easily be controlled by tailoring the fusion splicing parameters. Variations of this fabrication process consist in preconditioning the fibres tips, either with a liquid^14^, by arc discharge^15^, or employing a silica capillary tube^16^. Although these structures consist of hollow cavities within the fibres, they are mechanically strong, being resistant to lateral loads^17^ and strain^16^. These type of sensors can also withstand high temperatures since no film or polymer integrate the sensors structure^18^.

The salinity sensors developed in this work are based on optical fibre sensing elements consisting of optical cavities within the fibres. The cavities act as FP interferometers rendering the presence of a certain refractive index, which is detectable by a phase change of the interferometric response caused by the change of the optical path length of the cavity^19^. Employing single mode (SMF28e + , Corning) and multimode graded index fibres (GIF625, Thorlabs), a novel method for the fabrication of FP cavities within the optical fibre is presented, combining chemical etching and fusion splicing for the formation of a hollow cavity. The use of such structures as salinity sensors is demonstrated resulting in a sensitivity of 12.09 nm/M.

Vernier-effect based sensors have been demonstrated and applied to optical fibre-based FP sensors for measurement of strain, magnetic field and temperature^20,21^. Here, a configuration based on the Vernier effect is reported for salinity and temperature sensing. Such configuration is composed by a sensing FP along with a reference FP, which leads to a magnification of the sensitivity to salinity changes, where sensitivities as high as 82.61 nm/M are experimentally demonstrated, with the added advantage of measuring simultaneously temperature and salinity changes.

## Structure of the Sensors and Principle of Operation

### Individual configuration

**Figure 1:**
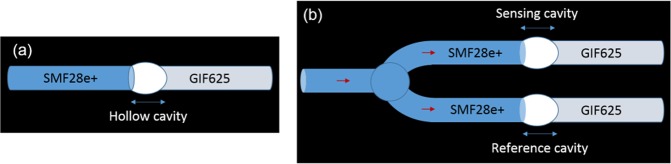
(**a**) Schematic of the sensing FP in single configuration, consisting of a single micro-bubble fabricated in-between the SMF28e+ and GIF625 fibres. (**b**) Schematic of the Vernier sensor: two cavities are employed and their spectra combined, where one is the sensing element and the other acts as a reference by being kept within a reference medium.

The fundamental sensing element consists of an individual air bubble fabricated in-between a section of single mode fibre (SMF) and graded index multimode fibre (GIF), as represented in Fig. 1(a). The hollow cavity is fabricated by a combination of hydrofluoric acid (HF) etching and fusion splicing, with ion milled microfluidic channels linking it to the surrounding medium.

The cavity acts as an FP interferometer, i.e., a cavity between two parallel reflective surfaces, where the reflective surfaces consist of the fiber-cavity interfaces. At the first interface, a fraction of the incident light is reflected while the remaining goes through it, propagating inside the cavity, and reaching the second surface. At this surface, part of the light is transmitted but another is reflected back onto the cavity, which will again propagate inside the cavity till it reaches the other surface, and then it reflects back again, and so on and so forth. The light rays inside the cavity interfere with each other depending on their relative path difference, so only certain frequencies are supported by the cavity. Consequently, the transmission and reflection spectra of an FP interferometer are composed of resonances whose spectral position depends on the optical path length of the cavity. In a round-trip in the FP interferometer, the phase difference (δ) between two waves is

1 $$\delta =\frac{4\,\pi \,n\,L}{\lambda }$$

where *n* is the effective RI of the mode propagating inside the cavity, *L* is the length of the cavity, and *λ* is the vacuum wavelength of the incident light. As such, the reflection spectrum (*R*) of an FP interferometer can be described by

2 $$R\propto 1-\frac{1}{1+F\,{\sin }^{2}(\delta /2)}$$

where *F* is the finesse coefficient. This coefficient is given by the following expression:

3 $$F=\frac{4\,{R}_{mirror}}{1+F\,{si}{{n}}^{2}(\delta /2)}$$

where *R*_*mirror*_ is the mirror reflection coefficient. From 3D-finite difference time domain simulations, the reflection coefficients of the fiber-cavity interfaces are approximately 3% at a wavelength of 1550 nm. Due to the low reflectivity value, the cavity acts as a low-finesse FP. Therefore, the FP can be described by a two-wave interferometer and, in this case, the reflection spectrum of the FP interferometer can be simply described by

4 $$R=A\,\cos (\frac{4\,\pi \,n\,L}{\lambda })$$

where A is the amplitude of the reflection spectrum.

By changing either the refractive index of the cavity, the resonances will shift. It is based on this principle that the interrogation and determination of salinity is possible using an FP interferometer: when the salinity varies, so does the RI, leading to a change in the optical length of the cavity which induces a shift of the fringes position.

### Vernier configuration

In the Vernier configuration, depicted in Fig. 1(b), the system is composed of a sensing FP and a reference FP, where the optical output of the two cavities is combined by an optical fibre circulator.

The sensing cavity is filled with the saline solution while the reference cavity is kept in air, acting as a reference medium with a constant RI. The cavities are designed to have slightly different optical lengths, and therefore have different free spectral ranges (FSRs), thus originating the so-called Vernier effect.

Through the Vernier effect, the sensitivity of a device is magnified. When the output of two mismatched interferometers is combined, the resulting spectrum consists of a series of resonances with different amplitudes. The amplitude modulation, or envelope, of the spectrum is a periodic signal with peaks and dips whose spectral position depend on the optical length of the composing cavities. When the optical length of the sensing cavity changes, the shift of the envelope, when compared to the spectral shift of the individual cavity, is magnified by a factor M due to the Vernier effect. The optical length change can be due to either a change in the physical length of the cavity or a change of the refractive index within the cavity.

Sensors that employ the Vernier effect can operate in two regimes. In the first regime, the free spectral range difference between the two resonators is large compared to the full width half maximum of the peaks of the individual interferometers, which originates the typical comb like spectrum. However, this regime has a limitation because the minimum detectable shift is equal to the free spectral range (FSR) of the individual sensor^22^. In the second regime, the FSR difference is smaller than the full width half maximum of the resonances of the individual interferometer. In this regime, a periodic envelope signal modulates the output of the sensor, overcoming the limitation of the sensors operating in the first regime^23,24^. The FP cavities employed in this work have low-finesse and small detune in terms of FSRs, therefore operating in the second regime.

So, when the output spectra of the two cavities with different optical lengths are added, the envelope of the reflection spectrum can be expressed by

5 $${R}_{E}=2\,A\,\cos (\frac{4\,\pi \,({n}_{R}\,{{\rm{L}}}_{R}-{n}_{S}{L}_{S})}{\lambda })$$

where L_R_ and L_S_ are the lengths of the reference and sensing cavities, and n_R_ and n_S_ are the refractive indices of the reference and sensing cavities, respectively. The period of R_E_ is^23^:

6 $$FS{R}_{E}=\frac{FS{R}_{R}\,FS{R}_{S}}{|FS{R}_{R}-FS{R}_{S}|}$$

where FSR_R_ is the free spectral range of the reference cavity, and FSR_S_ is the free spectral range of the sensing cavity. In such configuration, when there is a shift Δ(*nL*)_*S*_ in the sensing element, the shift of the sensor comprised by the sensing and reference FPs is magnified by a factor M^23^:

7 $$M=\frac{FS{R}_{R}}{|FS{R}_{R}-FS{R}_{S}|}$$

So, in comparison with a single sensing element, the sensitivity of the system composed by the sensing and reference elements will increase by a M-fold factor, as a small shift of the resonance wavelengths of the sensing interferometer will result in a much larger shift of the spectrum of the combined cavities. In addition, the Vernier configuration enables the simultaneous measurement of temperature and salinity, because the analysis can be performed by tracking both the shift of the envelope and the shift of the individual fringes.

## Fabrication Process

The formation of bubbles during fibre splicing is considered undesired in most applications since it creates a hollow cavity inside the fibre, which interrupts the fibre core and perturbs the transmission of the light. Bubble formation is usually due to a poor fibre profile (typically a poor cleave) or a fusion current that is too high. In this novel fabrication process presented here, the micro-bubble within the fibre is created by making use of both: first, a small cavity is etched by HF in a GIF, and then, the cavity is enlarged by fusion splicing the GIF to a SMF.

The first step of the fabrication process is the cleaving of the multimode graded index fibre tips (GIF625, Thorlabs), with a 62.5 μm core diameter and a numerical aperture of 0.275, which is then etched for 5 minutes in 9.6% HF gel, creating a shallow cavity in the fibre tip at an etch rate of 750 nm/min. The 9.6% HF buffered gel formulation, commonly used in dentistry as porcelain etchant gel, has a reduced etch rate compared to buffered hydrofluoric acid based etchants, providing better control of the etching depth and being at the same time safer to handle^25,26^.

**Figure 2:**
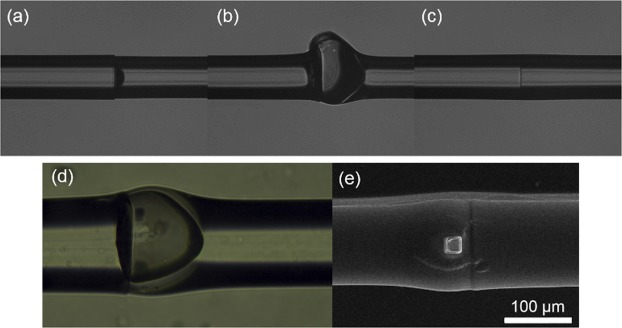
Top - Optical images of non-successful cavities formation due to (**a**) too low current, (**b**) excessively high current, and (**c**) no prior HF etch. Bottom - Images of hollow cavities fabricated by the hybrid method HF etch-fusion splicing: (**d**) optical microscope, and (**e**) scanning electron microscope.

Using a Fujikura 70 S splicer, the etched fibre tip is spliced to a cleaved single mode fiber (SMF28e^+^, Corning) employing the splicing parameters summarized in Table 1 that have been optimized to promote the formation of a hollow cavity during the splicing step. The fusion power is a key parameter in order to avoid unsuccessful micro-bubbles formation as depicted in Fig. 2(a,b): when the power is too low the bubble is not generated and only the small HF cavity is present; when the power is too high the bubble becomes too wide with thin fragile walls. Regarding the HF etching step, and as can be seen in Fig. 2(c), when no etching is performed in the fibre tip the micro-bubble does not form. An optical image of a micro-bubble successfully fabricated by the combined HF-fusion splicing process is shown in Fig. 2(d). The length of the micro-bubble can be controlled by changing the HF etching time: the length of the micro-bubbles is inversely proportional to the etching time, where cavities with lengths between 80 µm and 115 µm have been obtained. The sensing and reference cavities that are experimentally tested are 90 ± 3 μm and 102 ± 3 μm long, respectively, for an HF etching time of 5 and 2 minutes, respectively.

**Table 1:** Fusion splice parameters for fabrication of the hollow cavities using a Fujikura 70 S splicer.

SMF-GIF fusion splice parameters	
Cleaning Arc [ms]	OFF
Gap [μm]	15
Axial offset [μm]	30
Prefuse Time [ms]	OFF
Arc1 Power [bit]	STD-97
Arc1 Time [ms]	400
Fibre Type	MM-MM

The microfluidic access channels are obtained by focused ion beam milling and are roughly located in the centre of the cavity, which is easily located in the SEM as a bulge in the fibre next to the splice line. Two 15 × 15 μm^2^ channels are milled diametrically opposed to each other and their location is chosen to be roughly in the centre of the bubble, as shown in Fig. 2(e). The milling parameters are: I = 65 nA, V = 30 kV, resulting in a total milling time of approximately 8 minutes.

## Results and Discussion

### Methodology

**Figure 3:**
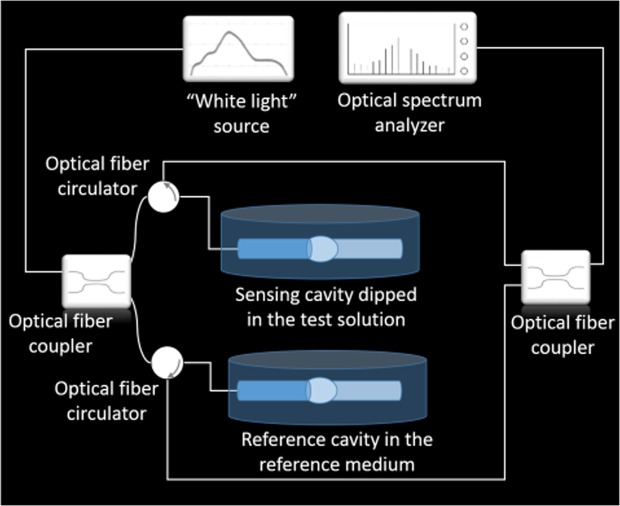
Schematic of the experimental setup.

The light is injected into the sensors by a broadband light test source (ASE730, Thorlabs), which operates in the wavelength range extending from 1530 nm to 1610 nm, while the output spectra are collected by an optical spectrum analyser (AQ6370C, Yokogawa). The use of an optical fibre circulator (6015-3-FC, Thorlabs) enables the measurement of the reflection outputs of the tested sensors. In Fig. 3 is depicted a schematic of the experimental setup for characterization of the Vernier-based sensor composed by the sensing and reference FPs. For the characterization of the individual configuration, i.e., the sensing architecture with no reference FP, the reference arm is disconnected.

To experimentally characterize the sensors sensitivity to salinity, the output of each sensor is acquired while the sensor is dipped in solutions with different salinity values. The aqueous solutions are prepared by dissolving sodium chloride, NaCl, in deionized (DI) water. The set of tested saline solutions has molar concentrations ranging from 0 M to 0.297 M, equivalent to a RI interval from 1.3176 to 1.3212.

For the experimental characterization of the sensors, a relatively large volume of solution is used to ensure that the salinity values do not vary in the cavity region as a consequence of evaporation of the aqueous solution throughout the procedure.

To avoid any cross-contamination between solutions of different salinity levels, the sensors are flushed with DI water in-between measurements. In addition, DI water is also used as a baseline to determine the wavelength shifts induced by the tested saline solutions. It is observed that for each cycle of flushing the sensors always recover to their baseline, proving the repeatability of the sensor. The recovery time is within three consecutive scans of the optical spectrum analyser, corresponding to approximately 15 seconds, which is a response time compatible with the possible application of these sensors.

The responsivity of the sensors to temperature changes is evaluated by dipping the sensors in a bath of DI water, whose temperature is regulated by a hot plate while the temperature reading values are recorded by a submerged digital thermometer.

Given that the FP sensing cavities are typically sensitive to axial strain and displacement, and in order to avoid cross-sensitivity, the sensors are immobilized with epoxy glue in a prototyped holder.

### Refractive index as a function of salinity

**Figure 4:**
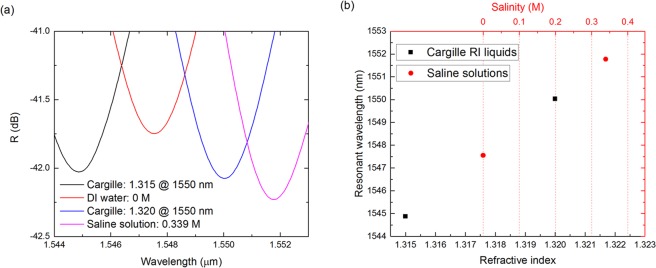
Resonant dips of the output spectrum of a single cavity dipped in two Cargille liquids with RI of 1.315 and 1.320 at 1550 nm, and in two saline solutions with salinity of 0 M and 0.339 M. (**a**) Detail of the output spectrum around 1550 nm. (**b**) Plot of the dip wavelength position as a function of RI, for the Cargille liquids, and salinity, for the saline solutions.

To establish the conversion between the RI values and salinity of the tested saline solutions at 1550 nm, it is necessary to resort to media of known RI at the wavelength of interest. Cargille refractive index liquids (Cargille Laboratories) have been selected, as these are very stable liquids with known RI values. An individual sensing FP is dipped in each of the two selected Cargille liquids, with RI of 1.315 and 1.320 at 1550 nm, respectively, from which the linear dependency of the dip’s spectral position as a function of RI can be obtained. The same sensing FP is also dipped in DI water (0 M) and a saline solution of 0.339 M, from which the dip position dependency on the salinity is extracted. The resonant dips in the region of 1550 nm for the tested liquids and saline solutions are shown in Fig. 4(a). The linear dependencies of the dip position as a function of salinity and as a function of RI, extracted from Fig. 4(b), are given by the following Equations:

8 $${\lambda }_{R}(S)=12.46\,n+1547.55$$

9 $${\lambda }_{R}(n)=1031.00\,n+189.11$$

where *λ*_*R*_ is the resonant wavelength in nm, *n* is the RI in RIU, and *S* is the salinity in M. In the region of 1550 nm and from the Equations (8,9), the relation between RI and salinity is given by

10 $$n\,(S)=\frac{12.45976\cdot {\rm{S}}-189.11}{1031.00}$$

### Single configuration

**Figure 5:**
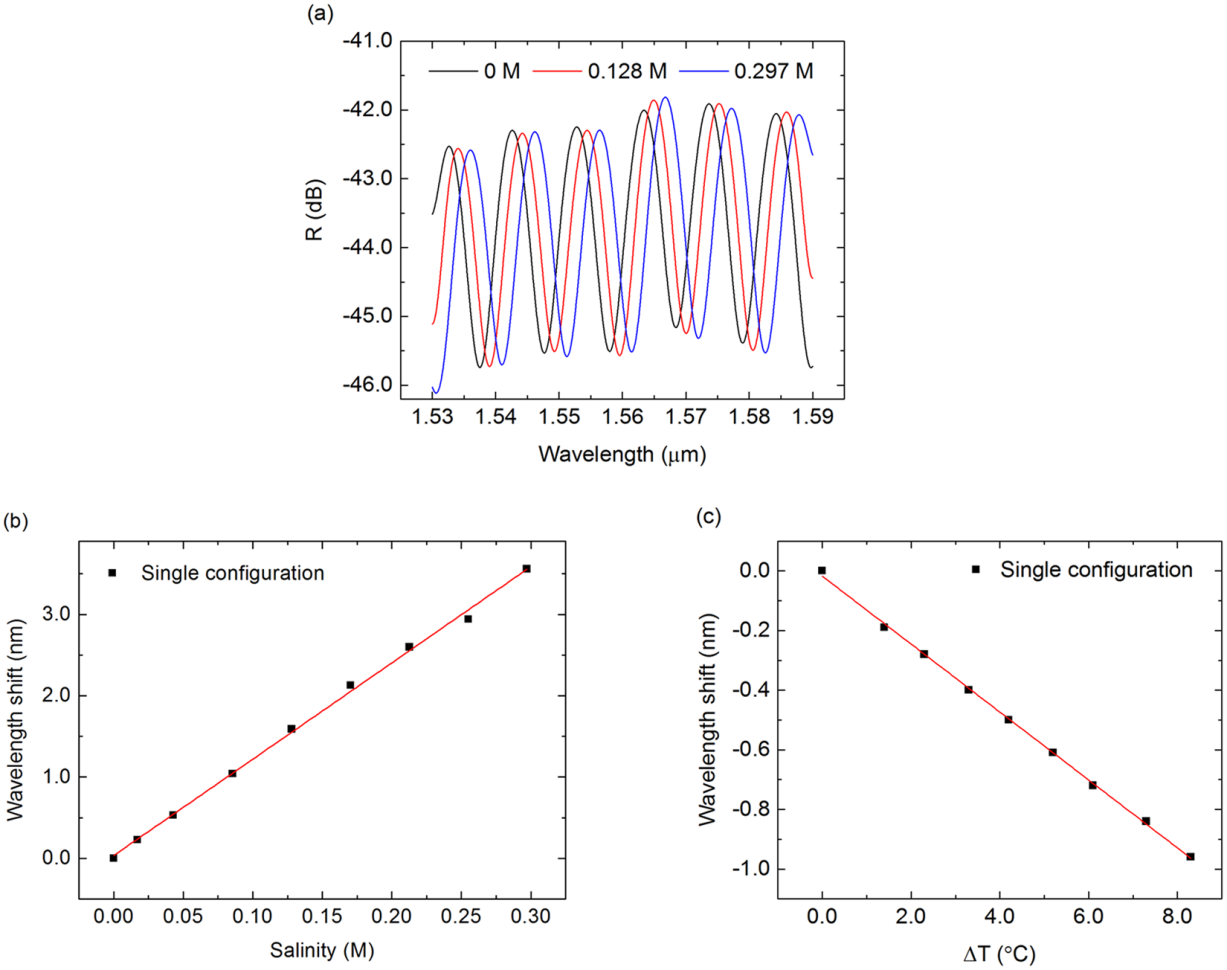
(**a**) Output spectra of a sensing FP in single configuration when immersed in three solutions of different salinity. Resonant wavelength shift at around 1550 nm of a single sensing cavity as a function of (**b**) salinity and (**c**) temperature shift.

The output spectra of the individual sensing FP for three different salinity values are plotted in Fig. 5(a). An individual spectrum consists of a set of interference fringes on the form of a sinusoidal wave, which is in good-agreement with a low-reflectivity FP interferometer, and therefore the output spectrum is similar to that of a two-wave interferometer.

When immersed in saline solutions of increasing salinity the resonant peaks of the sensing FP shift towards larger wavelengths, which is concordant with the increase of RI when the salinity increases.

The sensor’s sensitivity to salinity is interrogated by tracking the shift of the resonant wavelength. In Fig. 5(b), the shift of one of resonances at around 1550 nm is plotted as a function of the molarity of the saline solutions. A total shift of 3.56 nm is obtained for the salinity range from 0 M to 0.297 M. The sensitivity is obtained from a linear fit to the experimental data, yielding a value of 12.09 ± 0.20 nm/M with an R^2^ of 0.9978, for the single-FP configuration. Using the previously established conversion between RI and salinity, the resulting sensitivity to RI changes is 999.6 ± 16.5 nm/RIU.

The limit of detection of the sensor is extracted by acquiring 15 output spectra in series and then determining the variance of the position of the resonant wavelengths. The 3-sigma limit of detection, LOD_3σ_, is 2.1 × 10^−3^ M.

Regarding the temperature dependence of the sensor, which is plotted in Fig. 5(c), a total shift of −0.96 nm is obtained for a temperature shift of 8.3 °C (ranging from 21.7 °C to 30 °C). The sensitivity to temperature of the sensing FP is −115.3 ± 0.9 pm/°C, with an R^2^ of 0.9993, where the negative sign of the sensitivity arises from the negative thermo-optic coefficient of the water. The factor of 10^−4^ from the temperature to salinity sensitivities ratio is consistent with the thermo-optic coefficient of the water reported in the literature, in the order of 10^−4^ RIU/°C^27^. This sensor’s cross-sensitivity to temperature is very low because the temperature’s sensitivity is much lower than the sensitivity to salinity changes. However, in such configuration the simultaneous measurement of temperature and salinity shifts is not possible which have led to the development of the following configuration.

### Vernier configuration

**Figure 6:**
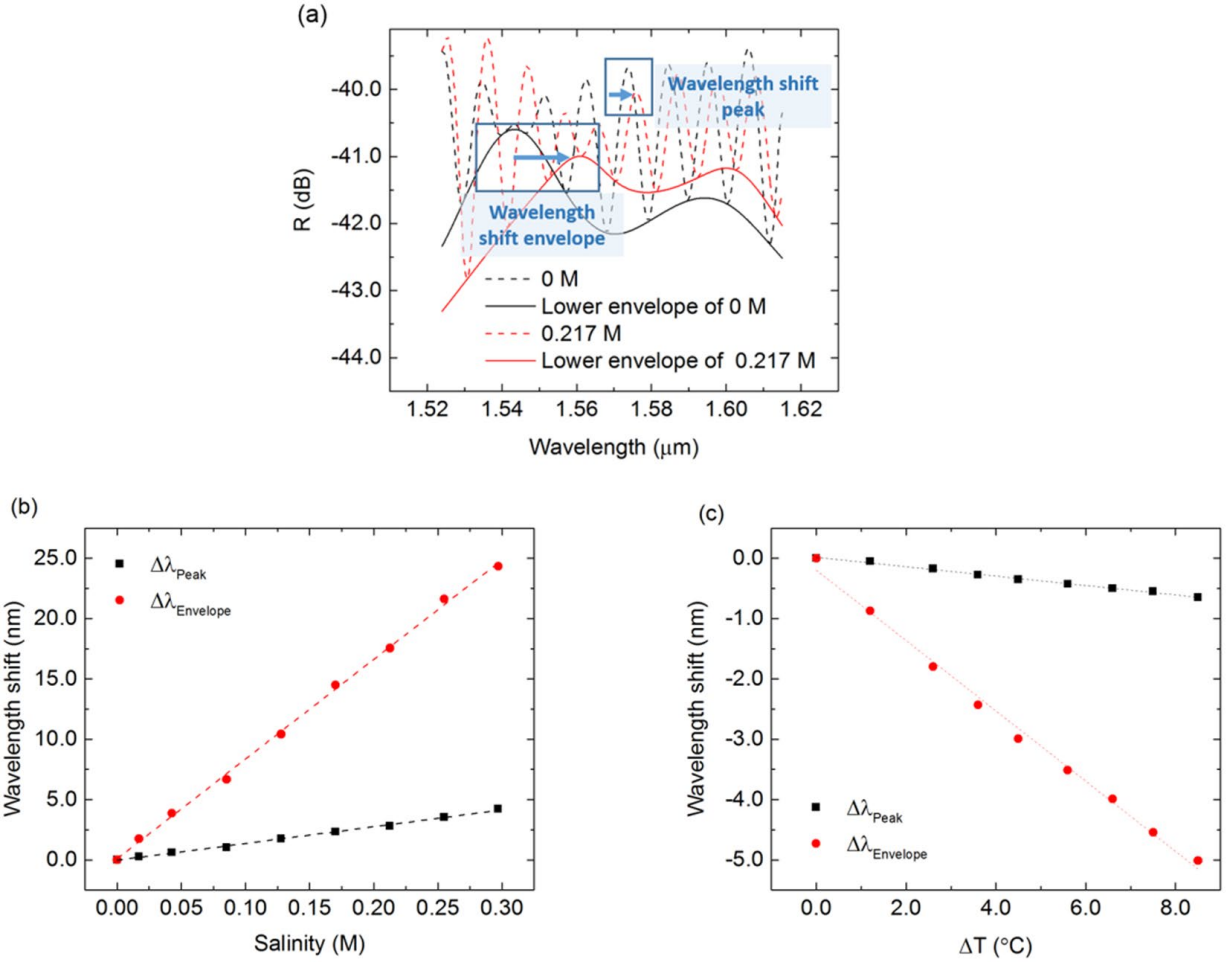
(**a**) Output spectra of the Vernier-configuration sensor comprised by the sensing and reference FPs in DI water (0 M) and in 0.217 M saline solution. Resonant wavelength shifts of the system comprised by the sensing and reference FPs as a function of (**b**) salinity and (**c**) temperature shift.

The characterized system is composed by the sensing FP, previously characterized in individual configuration, and a reference FP kept in air. The output of the sensor is plotted in Fig. 6(a), and it is composed by a set of interference fringes with modulated amplitude due to the difference of optical path lengths between the sensing and reference FPs. As described previously, the amplitude modulation, or envelope, is a function of the optical length of the cavities that comprise the system, and therefore its peaks position depends on the salinity and temperature of medium within the sensing cavity.

The shift of the envelope’s peak, Δ*λ*_Envelope_, as a function of the salinity level is plotted in Fig. 6(b), yielding a very high sensitivity to salinity changes of 82.61 ± 1.14 nm/M, or 6830.0 ± 94.3 nm/RIU, with an R^2^ of 0.9987, which is one of the highest sensitivities to salinity experimentally demonstrated by an optical fibre probe, to the best of the authors knowledge. The LOD_3σ_ for this sensor is 3.6 × 10^−3^ M.

The obtained sensitivity is 6.83-fold higher than the sensitivity of the sensing FP in single-configuration, 12.09 ± 0.20 nm/M, which demonstrates the magnification enabled by the Vernier configuration. For the cavities that compose this system, the expected analytical magnification given by Equation (7) is M = 7.14. The small discrepancy between magnification factors is justified by the uncertainty in the cavities length and in the RI of water. In theory, the detune in the FPs lengths could be designed to achieve a magnification factor as high as desired, but in practice that factor is limited because the envelope period cannot be larger than the wavelength range of both the light source and the optical spectrum analyser. Regarding the temperature effect, a sensitivity of −587.37 ± 0.01 pm/°C is obtained from Fig. 6(c) with an R^2^ of 0.9962.

Analysing the shift of the peak of the output spectra, the obtained sensitivity to salinity shifts is 13.91 ± 0.29 nm/M, or 1150.0 ± 24.0 nm/RIU, with an R^2^ of 0.9969, and to temperature changes is −0.0775 ± 0.0021 pm/°C, with an R^2^ of 0.9945.

### Sensitivity matrix for simultaneous measurement of temperature and salinity

For *in situ* sensing of salinity, the system needs to discriminate between temperature and salinity induced shifts, even when the sensitivity of the system to temperature shifts is much smaller than its sensitivity to salinity changes. As such, the system needs to be interrogated using two different methods, each yielding different sensitivities. As described previously, one of the methods consists in analysing the shift of the envelope of the output spectrum of the system, Δ*λ*_Envelope_, and the other method is based on the shift of the individual fringes of the output spectrum, Δ*λ*_Peak_. The wavelength shifts of the system’s output can be described as a function of salinity and temperature changes by

11 $$[\begin{array}{c}{\rm{\Delta }}{\lambda }_{Envelope}\\ {\rm{\Delta }}{\lambda }_{Peak}\end{array}]=[\begin{array}{cc}{S}_{Env,S} & {S}_{Env,T}\\ {S}_{Peak,S} & {S}_{Peak,T}\end{array}]\,[\begin{array}{c}{\rm{\Delta }}S\\ {\rm{\Delta }}T\end{array}]$$

where ΔS and ΔT are the water salinity and temperature variations, respectively. The matrix’s parameters S_m,n_, where m = Envelope or Peak, and n = Salinity (S) or Temperature (T), are the sensitivity coefficients obtained from the linear fits to the experimentally measured wavelength shifts reported in the previous section.

Inverting the matrix in Equation (11), the salinity and temperature shifts for the Vernier-configuration sensor are given by

12 $$[\begin{array}{c}{\rm{\Delta }}S\\ {\rm{\Delta }}T\end{array}]=0.0686[\begin{array}{cc}-0.0775 & 0.5874\\ -13.9111 & 82.6097\end{array}]\,[\begin{array}{c}{\rm{\Delta }}{\lambda }_{Envelope}\\ {\rm{\Delta }}{\lambda }_{Peak}\end{array}]$$

where ΔS is in M, ΔT is in °C, and Δ*λ* is in nanometres.

## Conclusions

In conclusion, the detection of salinity through hollow cavities fabricated by HF etching and fusion splicing has been demonstrated, resulting in high sensitivities in comparison with other optical fibre-based sensors. The sensitivity can be further increased when these structures are used in Vernier-configuration, where one of the cavities is kept in a reference medium, yielding very large sensitivities up to 82.61 nm/M or 6830.0 nm/RIU. Also, such configuration allows the simultaneous measurement of salinity and temperature which is an extremely important feature for *in situ* detection. The innovative fabrication process of the cavities is fairly simple and low-cost and it is based on two simple steps, etching by the HF gel and fusion-splicing, comprising the application of standard single mode and multimode graded index fibres. The characterization of the sensors was performed in the range of salinity between 0 M to 0.297 M, which widely covers the range of salinity found in the nature, from lakes, seas and oceans. Moreover, and even though the characterization of the reported sensors was performed in reflection, these sensors can be employed in transmission mode without loss of generality.

## Data Availability

The datasets generated during and/or analysed during the current study are available from the corresponding author on reasonable request.
