# Author Correction: Optical fibre Fabry-Pérot interferometer based on inline microcavities for salinity and temperature sensing

**DOI:** 10.1038/s41598-020-63856-1

**Published:** 2020-04-17

**Authors:** Raquel Flores, Ricardo Janeiro, Jaime Viegas

**Affiliations:** Department of Electrical and Computer Engineering, Khalifa University of Science and Technology, Masdar City Campus, Abu Dhabi, United Arab Emirates

Correction to: *Scientific Reports* 10.1038/s41598-019-45909-2, published online 02 July 2019

This Article contains typographical errors in the ‘Results and Discussion’ section under subheading ‘Refractive index as a function of salinity’, where equation (8)

“$${\lambda }_{R}(S)=12.46\,n+1547.55$$”

should read:

“$${\lambda }_{R}(S)=12.46\,S+1547.55$$”

and equation (10)

“$$n\,(S)=\frac{12.45976\cdot {\rm{S}}-189.11}{1031.00}$$ ”

should read:

“$$n\,(S)=\frac{12.46{\rm{S}}+1358.44}{1031.00}$$ ”.

